# Newer Classification System for Fissured Tongue: An Epidemiological Approach

**DOI:** 10.1155/2015/262079

**Published:** 2015-09-20

**Authors:** Ramachandran Sudarshan, G. Sree Vijayabala, Y. Samata, A. Ravikiran

**Affiliations:** ^1^Department of Oral Medicine and Radiology, Best Dental Science College and Hospital, Madurai 625104, India; ^2^Department of Dentistry, ESIC Medical College and PGIMSR, Ashok Pillar Road, KK Nagar, Chennai 600078, India; ^3^Department of Oral Medicine and Radiology, SIBAR Institute of Dental Sciences, Guntur 522509, India

## Abstract

*Introduction.* Fissured tongue is a commonly encountered tongue disorder in dental practice. But there is a lack of data on different pattern, severity, and association of fissuring with various systemic disorders and other tongue anomalies. This study attempts to establish a classification system for fissured tongue and to know the correlation with the systemic health and other disorders of the tongue. *Materials and Methods.* A total of 1000 subjects between the age groups of 10 and 80 years were included in the study. Pattern of fissuring, allied systemic diseases, and related tongue anomalies were tabulated. *Results.* Out of 1000 subjects, 387 subjects presented with fissured tongue. Out of 387 subjects, hypertension was present in 57 cases, 18 subjects had diabetes, and 3 subjects had both hypertension and diabetes. Central longitudinal type was found to be the most common type of tongue fissuring. *Conclusion.* Fissured tongue has been found to be associated with certain systemic disease and further researches are required to know positive correlation. If a correlation exists, such disorders could be diagnosed earlier by identifying fissured tongue at an earlier age.

## 1. Introduction

In the medical philosophies, the tongue has been believed to be an indicator of health for several decades. Customarily, the tongue is known to be a mirror of the oral and general health. Hippocrates, Galen, and others considered the tongue as the barometer of health [1].

In the oral mucosal disorders, tongue lesions constitute substantial proportions. Variable range of prevalence rates has been reported in the different regions. This difference may get along with racial factors, gender and systemic disorders, and so forth [2].

Fissured tongue is an inherited disorder manifested with grooves that can vary in size and depth. A definite etiology does not exist but a polygenic mode of inheritance is postulated. It is an incidental finding diagnosed during the routine intraoral examination. Usually the fissured tongue is asymptomatic unless entrapment of debris within fissure occurs [3].

The rationale of this present study is a preliminary novel attempt to derive a classification for fissured tongue based on the pattern of fissuring, number of fissures on the tongue, and associated symptoms if any. Further correlation of these findings with the systemic health was also done to find out if any relevance exists.

## 2. Materials and Methods

The present study was conducted at the Department of Oral Medicine and Radiology, SIBAR Institute of Dental Sciences, Guntur. A total of 1000 consecutive patients among the age groups of 10 to 80 years were included in the study. All the patients were accessible at the hospital for regular checkup and dental treatment. The study was conducted from February 2012 to April 2012 by a single investigator as the fissured tongue is routinely noted as a common finding on intraoral examination [3]. Acquiescence to do the study was obtained from institutional review board and informed consent to take photographs was given by the study subjects. Only those patients who gave the informed consent participated in the study. A detailed history regarding the demographic data, systemic health, and associated tongue symptoms was recorded. Medical history of the subjects was confirmed after evaluating their recent medical records. The subjects were seated on the dental chair and were examined using mouth mirror and straight probe and under illumination with dental chair light. Study subjects were asked to swish the mouth with sterile water before performing an intraoral examination of the tongue. The study subjects were asked to open the mouth and protrude the tongue as much as possible. The subjects were examined with sterile gloves and sterile gauze was used to hold the tip of the tongue to ease complete examination of the tongue. Clinical analysis regarding the pattern of fissuring of the tongue, number of fissures in tongue, and associated tongue anomalies was recorded. Statistical analysis was performed and a *P* value < 0.05 was considered to be statistically significant.

## 3. Results

Out of 387 subjects with fissured tongue, 235 (60.7%) were male subjects and 152 (39.3%) were female subjects.

Based on our observations, we have proposed a novel method of classification and classified fissured tongue as follows.

**Figure 1:**
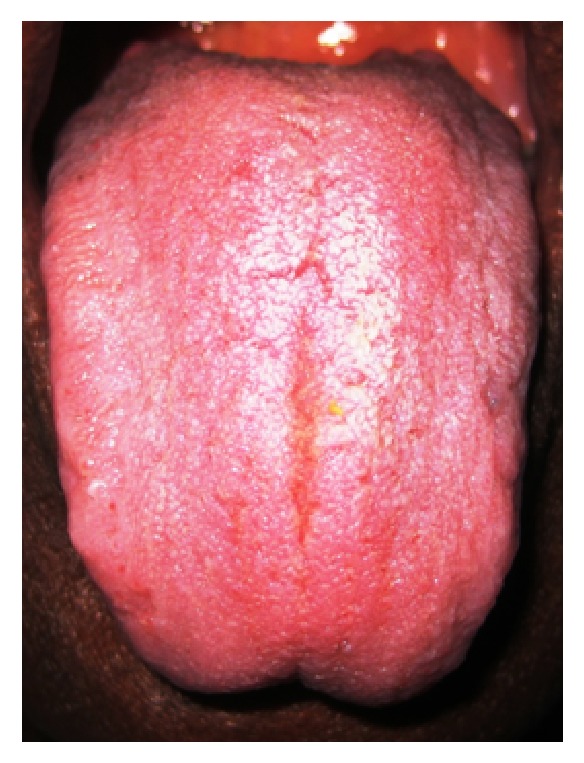
Central longitudinal and coated tongue.

**Figure 2:**
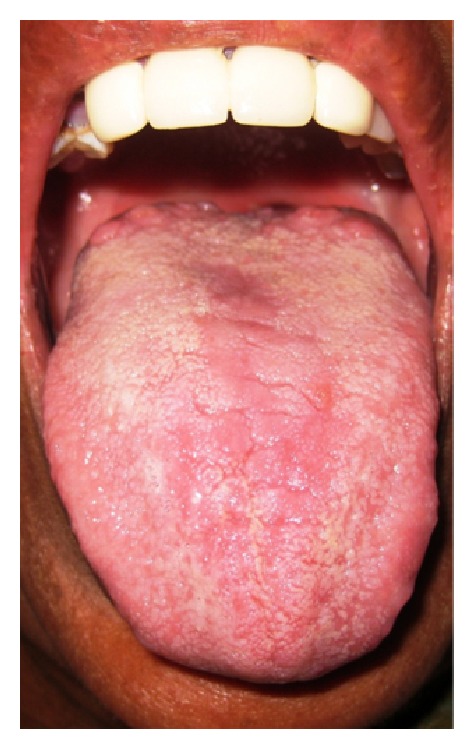
Central transverse type.

**Figure 3:**
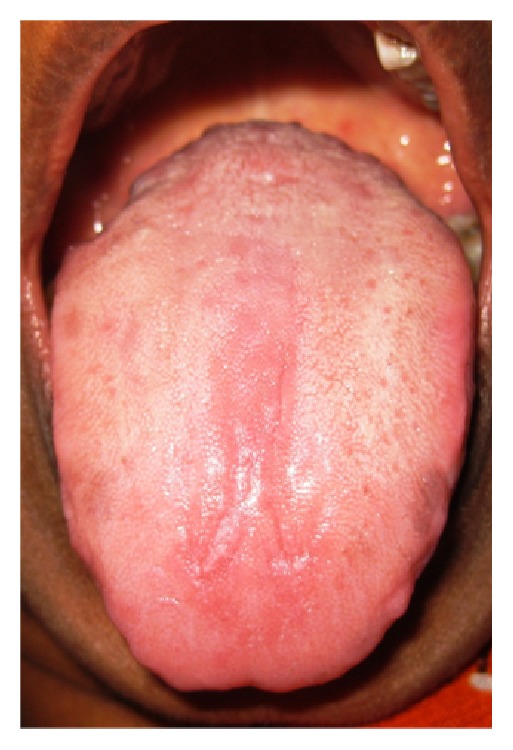
Lateral longitudinal type.

**Figure 4:**
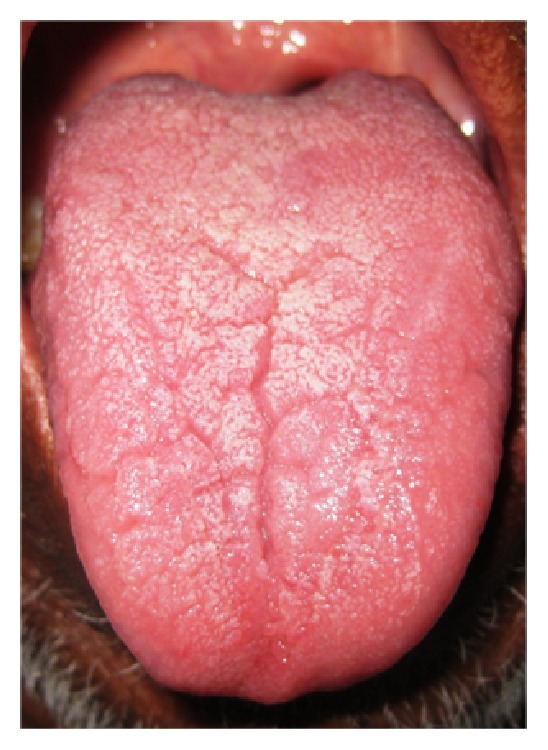
Branching type.

**Figure 5:**
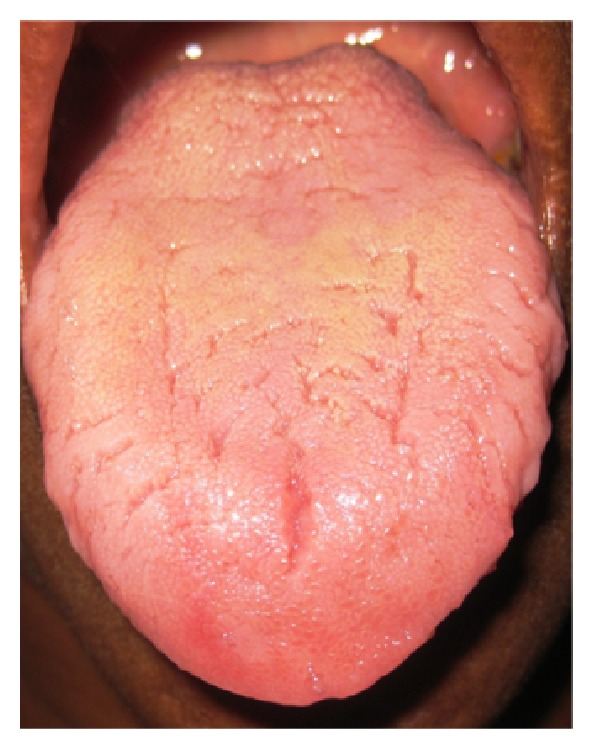
Diffuse type.

1. Based on pattern of tongue fissures.
  - Central longitudinal pattern in 196 (50.6%) subjects (Figure 1): vertical fissure running along the midline of the dorsal surface of the tongue.
  - Central transverse pattern in 42 (10.9%) subjects (Figure 2): horizontal fissure/fissures crossing the midline.
  - Lateral longitudinal pattern in 20 (5.2%) subjects (Figure 3): vertical fissure/fissures running laterally to the midline.
  - Branching pattern in 68 (17.6%) subjects (Figure 4): transverse fissures extending from the central longitudinal fissure (branching tree appearance).
  - Diffuse pattern in 61 (15.8%) cases (Figure 5): fissures diffusely distributed across the dorsal surface of the tongue.
2. Based on number of tongue fissures.
  - Mild: tongue fissures ranging from 1 to 3 in number.
  - Moderate: tongue with more than 3 fissures.
  - Severe: tongue with more than 10 fissures.
3. Based on associated symptoms such as burning sensation and feeling of food lodgement.
  - Without burning sensation.
  - With burning sensation.

The predominantly observed fissuring pattern was the central longitudinal pattern (50.6%) and the least observed pattern was the lateral longitudinal pattern (5.2%). Fissuring pattern and number of fissures were correlated with gender (Pearson Chi-Square is 0.528) results depicted that out of 387 subjects 235 were males and 152 were females.

Pattern of fissuring and number of fissures were correlated and the results showed that the majority of mild cases were associated with central longitudinal pattern (76.9%) followed by central transverse (15.3%) and lateral longitudinal (7.8%) patterns. In moderate group of cases, the majority belonged to branching type (95.7%) followed by central transverse type (4.3%). In the severe group of cases, the majority were of diffuse type (96.8%) followed by branching type (3.2%) (Fisher's Exact Test < 0.001).

The majority of patients with fissured tongue were asymptomatic. Out of these 22 subjects, 6 (27.3%) subjects were in central longitudinal and diffuse types, 4 (18.2%) subjects were in central transverse and branching types, and 2 (9.1%) subjects were in lateral longitudinal type.

Pattern of fissuring in patients was correlated with their medical history. Out of 387 subjects with fissured tongue, 289 subjects were without any medical disorder and the remaining 98 subjects had associated systemic history (Table 1). Hypertension was present in 57 subjects, 18 subjects had diabetes, and 3 subjects had both hypertension and diabetes. Several other systemic disorders also coexisted with the occurrence of fissured tongue. In our observation it included asthma, gastritis, trigeminal neuralgia, epilepsy, candidiasis, carcinoma, and Down's syndrome. Several associated tongue disorders have also been observed in the subjects (Table 2).

## 4. Discussion

Fissured tongue is believed to be a normal variant in fewer than 10% of the population and perhaps genetically oriented. The exact cause of fissured tongue is unidentified; nevertheless a polygenic mode of inheritance is alleged because the situation is seen clustering in families [4].

The asymptomatic fissured tongue is frequently perceived during intraoral examination as a subsidiary finding. Fissured tongue may be apparent at birth or become apparent during later stages with varying degree of depth from 2 to 6 mm [4, 5].

Gender based occurrence of fissured tongue was similar to a Libyan based adult population study that depicted that males were commonly affected [6]. But the findings were conflicting with the study conducted in Jordan that depicted that increased prevalence of fissured tongue in female subjects [2].

Fissured tongue has been discussed in the literature based on position as medial and lateral types. Several variations have been proposed in the presentation of grooves or furrows that are typically situated in the dorsolateral area of the tongue. The next pattern is a central fissure with several fissures branches at right angles from the central form. In the severe form, numerous fissures cover the entire dorsal surface dividing the tongue papillae into multiple separate “icelands” or lobules which correlates with our diffuse form of fissure patterning [7].

Burning sensation on the tongue may probably correlate with the systemic factors and poor oral hygiene. Local factors implicated in the etiology are ill fitting prosthesis, infection, parafunctional habits, allergic reaction, xerostomia and galvanism, and so forth [8]. Systemic factors concerned with burning sensation include medication, anemia, esophageal reflux, deficiency of vitamin B complex, zinc, iron, esophageal reflux, and psychological factors [9].

According to the literature, fissured tongue is usually asymptomatic. Few patients complain of mild pain. The circumstance is worsened by entrapment of food particles within the fissures and poor oral hygiene and nutrition [7].

A Hungarian epidemiological survey depicted that fissured tongue was coexistent with diabetes mellitus followed by hypertension [10]. But in our study, most of the systemically debilitated patients had hypertension followed by diabetes. Most of the hypertensive patients had central longitudinal pattern of fissuring which was found to be the common type in patients without any medical history. Reports suggest that an association exists between geographic tongue and fissured tongue [11, 12]. Contradicting to the previous reported studies, coated tongue was found to be more associated with fissured tongue rather than geographic tongue which was similar to the observation of the Jordanian study [2]. Fissured tongue was found to be associated with certain syndromes like Melkersson-Rosenthal syndrome, Coffin-Lowry syndrome, Fraser's Syndrome, Down's syndrome, Oral-Facial-Digital Syndrome Type I, Mohr Syndrome, Pierre Robin Syndrome, Maroteaux-Lamy Syndrome, ECC syndrome, and even Sjögren syndrome [13, 14].

## 5. Conclusion

This study portrays the newer classification for fissured tongue, its pattern, frequencies of pattern, associated symptoms, and coexisting systemic disorders. The association of fissured tongue with several systemic disorders has to be extensively studied in a larger population to validate its specific relation with systemic illness. Genetic preponderance of fissured tongue should also be extensively investigated. Further, if this genetic preponderance is substantiated in multicentre trials, fissured tongue diagnosed in early stages of life might be supportive of earlier diagnosis of systemic disorders.

## Figures and Tables

**Table 1:** Correlation between patterning of fissuring and medical history.

Medical history	Pattern of fissuring										Total	
	Central longi.		Central trans.		Branching		Diffuse		Lateral longi.			
	*N*	%	*N*	%	*N*	%	*N*	%	*N*	%	*N*	%
Hypertension (HTN)	20	35.1	15	26.3	4	7.0	14	24.6	4	7.0	57	100.0
Diabetes Mellitus (DM)	2	11.1	2	11.1	7	38.9	7	38.9	0	0.0	18	100.0
Gastritis	4	44.4	0	0.0	3	33.3	2	22.2	0	0.0	9	100.0
Asthma	0	0.0	3	75.0	1	25.0	0	0.0	0	0.0	4	100.0
Trigeminal neuralgia	0	0.0	0	0.0	1	100.0	0	0.0	0	0.0	1	100.0
Syndrome associated	0	0.0	0	0.0	2	100.0	0	0.0	0	0.0	2	100.0
Epilepsy	0	0.0	0	0.0	0	0.0	1	100.0	0	0.0	1	100.0
HTN + DM	3	100.0	0	0.0	0	0.0	0	0.0	0	0.0	3	100.0
HTN + gastritis	2	100.0	0	0.0	0	0.0	0	0.0	0	0.0	2	100.0
Candidiasis + carcinoma	1	100.0	0	0.0	0	0.0	0	0.0	0	0.0	1	100.0
No medical history	164	56.7	22	7.6	50	17.3	37	12.8	16	5.5	289	100.0
Total	196	50.6	42	10.9	68	17.6	61	15.8	20	5.2	387	100.0

Fisher's Exact Test (<0.001).

**Table 2:** Correlation between pattern of fissuring and other tongue disorders.

Other tongue disorders	Pattern of fissuring										Total	
	Central longi.		Central trans.		Branching		Diffuse		Lateral longi.			
	*N*	%	*N*	%	*N*	%	*N*	%	*N*	%	*N*	%
No tongue disorders	188	53.4	37	10.5	63	17.9	48	13.6	16	4.5	352	100.0
Coated tongue	3	18.8	2	12.5	3	18.8	5	31.3	3	18.8	16	100.0
Oral submucous fibrosis	2	66.7	0	0.0	0	0.0	1	33.3	0	0.0	3	100.0
Depapillated tongue	1	33.3	2	66.7	0	0.0	0	0.0	0	0.0	3	100.0
Hairy tongue	0	0.0	0	0.0	0	0.0	1	100.0	0	0.0	1	100.0
Crenated tongue	0	0.0	0	0.0	1	50.0	0	0.0	1	50.0	2	100.0
Ankyloglossia	1	100.0	0	0.0	0	0.0	0	0.0	0	0.0	1	100.0
Lichen planus (reticular)	0	0.0	1	100.0	0	0.0	0	0.0	0	0.0	1	100.0
Geographic tongue	0	0.0	0	0.0	1	33.3	2	66.7	0	0.0	3	100.0
Erythema	1	100.0	0	0.0	0	0.0	0	0.0	0	0.0	1	100.0
Coated tongue + crenated tongue	0	0.0	0	0.0	0	0.0	1	100.0	0	0.0	1	100.0
Coated tongue + leukoplakia	0	0.0	0	0.0	0	0.0	1	100.0	0	0.0	1	100.0
Coated tongue + OSMF + depapillated tongue	0	0.0	0	0.0	0	0.0	1	100.0	0	0.0	1	100.0
Depapillated tongue + angular cheilitis	0	0.0	0	0.0	0	0.0	1	100.0	0	0.0	1	100.0
Total	196	50.6	42	10.9	68	17.6	61	15.8	20	5.2	387	100.0

Fisher's Exact Test (0.001).
